# Nicotinic α7 receptor activation selectively potentiates the function of NMDA receptors in glutamatergic terminals of the nucleus accumbens

**DOI:** 10.3389/fncel.2014.00332

**Published:** 2014-10-16

**Authors:** Stefania Zappettini, Massimo Grilli, Guendalina Olivero, Jiayang Chen, Cristina Padolecchia, Anna Pittaluga, Angelo R. Tomé, Rodrigo A. Cunha, Mario Marchi

**Affiliations:** ^1^Faculté de Médecine, Institut de Neurosciences des Systèmes Inserm UMR1106, Aix Marseille Université La TimoneMarseille, France; ^2^Department of Pharmacy, University of Genoa, Viale CembranoGenoa, Italy; ^3^Center of Excellence for Biomedical Research, University of GenoaGenoa, Italy; ^4^CNC-Center for Neuroscience and Cell Biology, University of CoimbraCoimbra, Portugal; ^5^Department of Life Sciences, Faculty of Sciences and Technology, University of CoimbraCoimbra, Portugal; ^6^Faculty of Medicine, University of CoimbraCoimbra, Portugal

**Keywords:** nicotinic receptors, NMDA receptors, nicotine treatment, neurotransmitters release, synaptosomes, nucleus accumbens

## Abstract

We here provide functional and immunocytochemical evidence supporting the co-localization and functional interaction between nicotinic acetylcholine receptors (nAChRs) and N-methyl-D-aspartic acid receptors (NMDARs) in glutamatergic terminals of the nucleus accumbens (NAc). Immunocytochemical studies showed that a significant percentage of NAc terminals were glutamatergic and possessed GluN1 and α7-containing nAChR. A short-term pre-exposure of synaptosomes to nicotine (30 µM) or choline (1 mM) caused a significant potentiation of the 100 µM NMDA-evoked [^3^H]D-aspartate ([^3^H]D-Asp) outflow, which was prevented by α-bungarotoxin (100 nM). The pre-exposure to nicotine (100 µM) or choline (1 mM) also enhanced the NMDA-induced cytosolic free calcium levels, as measured by FURA-2 fluorescence imaging in individual NAc terminals, an effect also prevented by α-bungarotoxin. Pre-exposure to the α4-nAChR agonists 5IA85380 (10 nM) or RJR2429 (1 µM) did not modify NMDA-evoked ([^3^H]D-Asp) outflow and calcium transients. The NMDA-evoked ([^3^H]D-Asp) overflow was partially antagonized by the NMDAR antagonists MK801, D-AP5, 5,7-DCKA and R(-)CPP and unaffected by the GluN2B-NMDAR antagonists Ro256981 and ifenprodil. Notably, pre-treatment with choline increased GluN2A biotin-tagged proteins. In conclusion, our results show that the GluN2A-NMDA receptor function can be positively regulated in NAc terminals in response to a brief incubation with α7 but not α4 nAChRs agonists. This might be a general feature in different brain areas since a similar nAChR-mediated bolstering of NMDA-induced ([^3^H]D-Asp) overflow was also observed in hippocampal synaptosomes.

## Introduction

Adaptive changes in the glutamatergic inputs triggering information processing in the nucleus accumbens (NAc) are increasingly recognized as key features underlying mood dysfunction and addiction (Carlezon and Thomas, 2009; Reissner and Kalivas, 2010). In particular N-methyl-D-aspartic acid receptors (NMDARs) play a critical role in these adaptive changes (Ma et al., 2009), which are modulated by the cholinergic system, namely through nicotinic acetylcholine receptors (nAChRs; Giocomo and Hasselmo, 2007; Timofeeva and Levin, 2011). These two signaling systems are intertwined as heralded by the ability of nicotine to modulate both the subunit composition (Delibas et al., 2005; Levin et al., 2005; Wang et al., 2007) and several functions of NMDAR (Yamazaki et al., 2006; Liechti and Markou, 2008; Lin et al., 2010; Li et al., 2013; Ávila-Ruiz et al., 2014; Callahan et al., 2014; Salamone et al., 2014). This interaction between nAChR and NMDAR seems most evident in nerve terminals (Lin et al., 2010; Salamone et al., 2014): this is of particular interest in view of the increasingly recognized role of presynaptic NMDARs in the control of synaptic plastic changes in different brain areas (Sjöström et al., 2003; Corlew et al., 2008; Bidoret et al., 2009). Thus, we now combined immunological, pharmacological and neurochemical approaches applied to purified nerve terminals to study NMDAR function in glutamatergic terminals in the NAc and we tested whether these presynaptic NMDARs were controlled by nAChRs.

## Materials and methods

### Animals and brain tissue preparation

Adult male rats (Sprague–Dawley, 200–250 g) were housed at constant temperature (22 ± 1°C) and relative humidity (50%) under a regular light–dark schedule (light 7.00 a.m.–7.00 p.m.) with food and water freely available. The experimental procedures were approved by the Ethical Committee of the Pharmacology and Toxicology Section (University of Genoa) (protocol number 124/2003-A), in accordance with the Italian and European legislation on animal experimentation (2010/63/EU). All efforts were made to minimize animal suffering and to use the minimal number of animals required to produce reliable results.

### Preparation of synaptosomes

Synaptosomes were prepared essentially as previously described (Grilli et al., 2008, 2009). Rats were killed by decapitation, their brains were rapidly removed at 0–4°C and dissected to collect the NAc (sections between Bregma 0.7–2.2 mm), according to the atlas of Paxinos and Watson (1986), or the hippocampus. The tissue was homogenized in 40 volumes of 0.32 M sucrose, buffered to pH 7.4 with phosphate (final concentration 0.01 M). The homogenate was centrifuged at 1000 *g* for 5 min, to remove nuclei and cellular debris, and crude synaptosomes were isolated from the supernatant by centrifugation at 12,000 *g* for 20 min. The synaptosomal pellet was then resuspended in Krebs medium with the following composition (mM): NaCl 128, KCl 2.4, CaCl_2_ 3.2, KH_2_PO_4_ 1.2, MgSO_4_ 1.2, HEPES 25, glucose 10, pH 7.2–7.4. The purification of nerve terminals for calcium imaging and immunocytochemical assays was carried out using a sucrose/Percoll fractionation, as previously described (Rodrigues et al., 2005).

### Neurotransmitter release

The release of glutamate was gauged using the non-metabolizable tracer [^3^H]D-aspartate ([^3^H]D-Asp), which was loaded by incubation of the synaptosomes for 20 min at 37°C with 0.08 µM [^3^H]D-Asp. Identical samples of the synaptosomal suspension were then layered over microporous filters at the bottom of parallel superfusion chambers thermostated at 37°C and the synaptosomes were superfused with a flow rate of 0.5 mL/min with Krebs medium. After 36 min (*t* = 36 min), four consecutive 3-min fractions of the eluent were collected. Synaptosomes were then exposed to NMDAR agonists (100 µM NMDA and 10 µM glycine) or to depolarizing agent (4-aminopyridine, 4AP, 10 µM) from *t* = 39 min onwards, while antagonists were present from 8 min before addition of the agonists onwards. Exposure to nAChR agonists was done at *t* = 29 min for 10 min in absence or in presence of nAChR antagonists. The superfusate samples and the synaptosomes were then counted for radioactivity. Agonist effects were expressed as percent of the induced outflow over basal outflow, upon subtraction of the radioactivity released in the four fractions collected under basal condition (no drug added) from that released in presence of the stimulus.

### Calcium imaging

Purified nerve terminals (500 µg of protein) were resuspended in 1 mL of HEPES-buffered medium (HBM with 122 mM NaCl, 3.1 mM KCl, 0.4 mM KH2PO4, 5 mM NaHCO3, 1.2 mM MgSO_4_, 10 mM HEPES, 10 mM glucose, pH 7.4). They were loaded with FURA-2 through incubation with HBM supplemented with 5 µM FURA-2-AM, 0.02% pluronic acid F-127, 0.1% bovine serum albumin (BSA, fatty-acid free) and 1.33 mM CaCl_2_ for 1 h at 25°C and then allowed to attach onto poly-D-lysine-coated coverslips. The terminals were washed with HBM containing 1.33 mM CaCl_2_ and mounted in a small superfusion chamber (RC-20; Warner Instruments, Harvard, UK) on the stage of an inverted fluorescence microscope (Axiovert 200; Carl Zeiss, Jena, Germany).

Nerve terminals were alternately excited with UV light centered at 340 and 380 nm using an optical splitter (Lambda DG4; Sutter Instruments, Novato, CA, USA), with an exposure time of 2360 ms, and the emitted fluorescence images were captured through a 40× oil objective and a 510 nm band-pass filter (Carl Zeiss) connected to a digital camera (Cool SNAP; Roper Scientific, Trenton, NJ, USA). Results were expressed by plotting the time course of the ratio, *R*, of the average fluorescence light intensity emitted by a small elliptical region inside each terminal upon alternated excitation at 340 and 380 nm (*R* = F340/F380).

Increases in R correspond to increases of the levels of cytosolic free calcium, [Ca^2+^] (Lev-Ram et al., 1992; Castro et al., 1995). The basal ratio was measured during 60 s (i.e., 12 cycles) before stimulating the nerve terminals by superfusion with NMDA (100 µM) + glycine (10 µM) for 60 s. To measure the effects of the pre-treatment with different agonists and antagonists, nicotine (100 µM), 5IA85380 (10 nM), choline (1 mM) and α-bungarotoxin (10 nM) were added 1 min before the stimulus. A 30 s pulse of KCl (25 mM) was applied at the end of each experiment to confirm the viability of the studied nerve terminals. Changes in Calcium response were measured as ΔR, subtracting the baseline (before the drug stimulation) to the peak (after the drug stimulation). All tested compounds were prepared in HBM medium lacking Mg^2+^ ions to disclose the NMDA receptor-mediated effect, and they were added to the superfused nerve terminals, through a pressurized fast-exchange solution delivery system (AutoMate Scientific, Berkeley, CA, USA), with constant gassing of all superfusion solution with 95% O_2_/5% CO_2_.

### Immunocytochemical assays

Nerve terminals (500 µg of protein) were resuspended in 1 mL of phosphate-buffered saline (PBS, composed of 137 mM NaCl, 2.6 mM KCl, 1.5 mM KH_2_PO_4_, 8.1 mM Na_2_HPO_4_, pH 7.4) and allowed to attach onto poly-D-lysine-coated coverslips. The follow-up immuno-characterization of the nerve terminals used in FURA-2 fluorescence imaging experiments required the use of grid-etched glass coverslips. The platted nerve terminals were fixed with 4% (w/v) paraformaldehyde for 15 min, washed twice with PBS, permeabilized in PBS with 0.2% Triton X-100 for 10 min, and then blocked for 1 h in PBS with 3% BSA and 5% normal horse serum and washed twice with PBS. Triplicate coverslips from each sample were incubated at 25°C for 1 h and the primary antibodies were diluted in PBS with 3% BSA and 5% normal horse serum: mouse anti-GluN1 (1:500), guinea pig anti-vGLUT (1:1000), rabbit anti-α7 nAChR (1:500), rabbit anti-α4 nAChR (1:500). After three washes with PBS containing 3% BSA and 3% normal horse serum, the nerve terminals were incubated for 1 h at room temperature with AlexaFluor-594 (red)-labeled goat anti-rat IgG secondary antibodies (1:200) together with Alexa Fluor-488 (green)-labeled donkey anti-rabbit and with Alexa Fluor-350 (blue)-labeled donkey anti-mouse IgG secondary antibodies (1:200). We confirmed that the secondary antibodies only yielded a signal in the presence of the adequate primary antibodies and that the individual signals obtained in double-labeled fields were not enhanced over the signals obtained under single-labeling conditions. After washing and mounting onto slides with Prolong Antifade, the preparations were visualized in a Zeiss Axiovert 200 inverted fluorescence microscope equipped with a cooled CCD camera and analyzed with AxioVision software (version 4.6). Each coverslip was analyzed by counting three different fields containing a minimum of 500 elements each.

### Biotinylation and immunoblotting

Synaptosomes from the NAc of two rats were re-suspended in HBM at 4°C. The cell surface density of GluN2A was evaluated by performing surface biotinylation followed by immunoblots analysis, as previously described (Ciruela et al., 2006), with minimal modications. The synaptosomes were divided into two aliquots (500 µg protein each) and both were incubated for 10 min at 37°C under mild shaking; one aliquot was then treated for 10 min with 1 mM choline (T) while the other was kept as control (C). Choline exposure was terminated by dilution in cold washing buffer composed of 150 mM NaCl, 1 mM EDTA, 0.2% BSA, 20 mM Tris, pH 8.6. After washing twice in ice-cold washing buffer, the synaptosomes were labeled with 2 mg/ml of sulfo-NHS-SS-biotin in PBS with 1.5 mM MgCl_2_ and 0.2 mM CaCl_2_, pH 7.4 (PBS/Ca-Mg) for 1 h at 4°C. The biotinylation reaction was stopped by incubating with 1 M NH_4_Cl for 15 min at 4°C, followed by two washes with ice cold 100 mM NH_4_Cl in PBS/Ca-Mg, to quench biotin. Subsequently, biotinylated synaptosomes were lysed in RIPA buffer (500 µL) composed of 150 mM NaCl, 1 mM EDTA, 0.1% SDS, 1% Triton X-100, 1% sodium deoxycholate, 1 mM orthovanadate, protease inhibitor cocktail and 10 mM Tris, pH 7.4. The lysate was centrifuged at 20,000 × *g* for 10 min at 4°C, and samples (100 µg) were incubated with streptavidin magnetic beads (40 µL) for 1 h at room temperature under shaking. Biotinylated proteins, linked to streptavidin magnetic beads, were then added to pulled-down by exposure of the mixture to a magnetic field. After extensive washes, 1 × SDS-PAGE buffer was added and samples were boiled for 5 min at 95°C. Proteins were then loaded and electrophoretically separated on a 10% sodium dodecyl sulfate-PAGE gel and then transferred to PVDF membranes and probed for the proteins of interest by incubation with rabbit anti-GluN2A (1:2,000) or mouse anti-β -actin (1:10,000) primary antibodies for 1 h at room temperature with Tween 20-containing Tris-buffered saline (t-TBS), composed of 150 mM NaCl, 0.1% Tween 20, 5% non-fat dried milk and 20 mM Tris, pH 7.4. After washing, membranes were incubated for 1 h at room temperature with the appropriate horseradish peroxidase-linked secondary antibody (1:20,000), and immunoblots were visualized with an ECL (enhanced chemiluminescence) Plus Western blotting detection system. GluN2A subunit density was determined in the total synaptosomal lysate (Syn) and in the streptavidin-pulled-down fraction of control and choline-pretreated biotinylated synaptosomes (Ctr and Ch, respectively).

### Data analysis

Statistical comparison of the results was carried out using a Student’s *t-*test for independent means (for single pairs comparison); multiple comparisons were performed with one- or two-way ANOVA followed by Tukey-Kramer *post hoc* test. Values are expressed as means ± SEM and are considered significant for *p* < 0.05.

### Materials

[2,3-^3^H]D-aspartate (specific activity 11.3 Ci/mmol) was from Perkin Elmer (Boston, MA, USA); nicotine hydrogen tartrate salt, 4-aminopyridine (4-AP), N-methyl-D-aspartate (NMDA), fatty-acid free BSA, anti-β-actin monoclonal mouse IgG1, horseradish peroxidase-conjugated anti-mouse and anti-rabbit secondary antibodies and the protease inhibitor cocktail were from Sigma-Aldrich (St. Louis, MO, USA); 5-iodo-A-85380, ifenprodil, Ro256981, 5,7-dicholoro-kynuremic acid (DCKA), D-AP5, MK-801, (R)-CPP and RJR-2403 oxalate were from Tocris (Bristol, UK); FURA-2 AM, pluronic acid F-127 were performed by Molecular Probes, Leiden, Netherlands. β-actin monoclonal mouse IgG1, horseradish peroxidase-conjugated anti-mouse and anti-rabbit secondary antibodies protease inhibitor cocktail were obtained from Sigma Chemical Co. (St. Louis, MO, USA). Sulfo-NHS-SS-biotin and Streptavidin 14. Magnetic Beads were purchased from Pierce Thermo Scientific (Rockford, IL, USA), Western blotting detection system was purchased from GeHealthcare (Italy). Guinea pig anti-vGLUT, mouse anti-GluN1, AlexaFluor-594 (red)-labeled goat guinea pig IgG, Alexa Fluor-488 (green)-labeled donkey anti-rabbit, Alexa Fluor-350 (blue)-labeled donkey anti-mouse, secondary antibodies were from Invitrogen. Rabbit anti-α4 nAChR (1:500), rabbit anti-α7 nAChR and anti-rabbit polyclonal GluN1 antibody was from Chemicon International (Millipore, Billerica, MA, USA).

## Results

### Co-localization and functional interaction of nAChR and NMDAR in glutamatergic terminals of the rat nucleus accumbens

**Figure 1:**
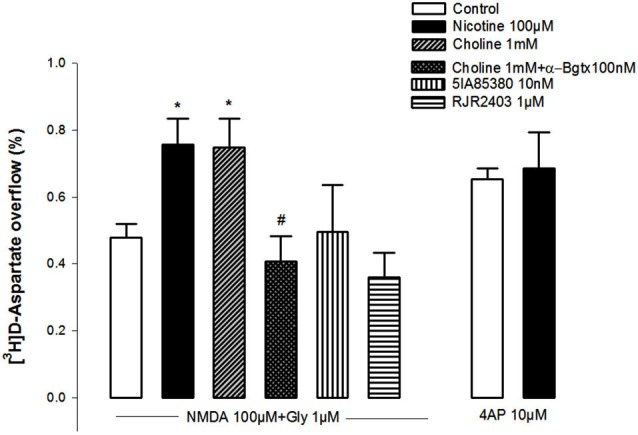
**Impact of the pre-treatment during 10 min with different nAChR agonists on the ability of NMDAR agonists (100 µM NMDA and 10 µM glycine) and of 4-AP to trigger [^3^H]D-Asp from rat NAc nerve terminals**. Data are means ± SEM of at least five experiments run in triplicate. *p < 0.05 vs. control; ^#^*p* < 0.05 vs. pretreatment with choline.

Figure 1 shows that NMDA (100 µM, plus 1 µM glycine) triggered the release of [^3^H]D-Asp from pre-labeled NAc synaptosomes. A 10 min pre-exposure of the synaptosomes to nicotine (100 µM) or choline (1 mM) significantly potentiated the NMDA-induced [^3^H]D-Asp outflow (+58%, and +56%, respectively). This potentiation was abolished in synaptosomes pretreated with the selective α7 nAChR antagonist α-bungarotoxin (100 nM; Figure 1). In contrast, the pre-exposure of the synaptosomes to the selective α4-nAChR agonist 5IA85380 (10 nM) or RJR2403 (1 µM) did not modify the NMDA-induced [^3^H]D-Asp outflow. It should be noted that the pre-treatment of NAc synaptosomes with nicotine failed to modify the 4-AP-induced [^3^H]D-Asp outflow (Figure 1).

**Figure 2:**
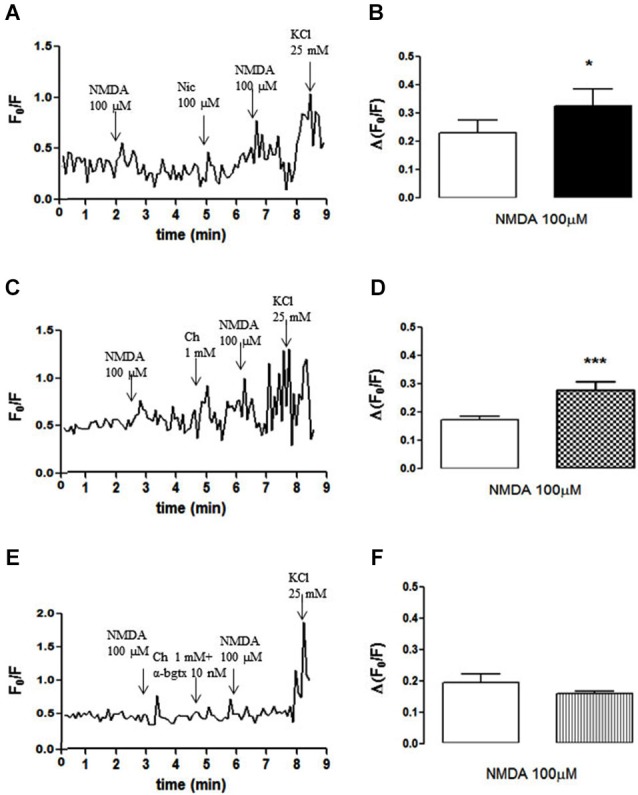
**(A, C, E)** Time course of FURA-2 fluorescence emission in individual nerve terminals from the rat NAc, which were challenged twice with NMDAR agonists (100 µM NMDA and 10 µM glycine), before and 60 s after pre-treatment with either 100 µM nicotine **(A)**, 1 mM choline **(C)** or 1 mM choline together with 10 nM α-bungarotoxin **(E)**. **(B, D, F)** Comparison of the average modification of calcium transients caused by NMDA agonists before (open bars) and 60 s after (filled bars) the exposure to 100 µM nicotine **(B)**, 1 mM choline **(D)** or 1 mM choline together with 10 nM α-bungarotoxin **(F)**. Drugs were applied for 60 s, at the end of the wash out of the previous application and the arrows identify the peaks. Values are mean ± SEM of at least four experiments *p < 0.05 and ***p < 0.001, using a paired Student’s *t* test.

The amplitude of the NMDA (100 µM, plus 10 µM glycine)-induced increase in cytosolic free calcium in individual NAc terminals (Figures 2A,B) was also potentiated by pre-exposure to nicotine (100 µM; Figures 2A,B) or choline (1 mM; Figures 2C,D), an effect that was blunted by α-bungarotoxin (10 nM; Figures 2E,F). These observations provide further evidence that the activation of α7-containing nAChR bolsters NMDAR-mediated functions in NAc synaptosomes.

**Figure 3:**
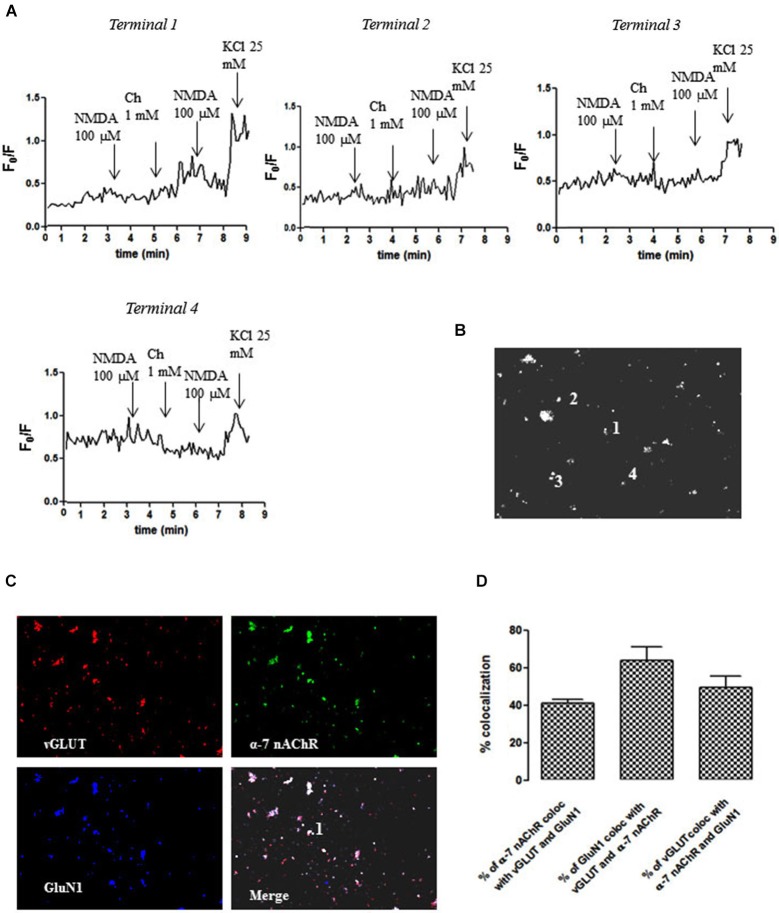
**Different impact of choline (1 mM) pre-treatment on the ability of NMDAR agonists to trigger calcium transients in different individual terminals from the rat NAc**. **(A)** Time course of FURA-2 fluorescence emission in different individual nerve terminals (terminal 1–terminal 4), which were challenged twice with NMDAR agonists (100 µM NMDA and 10 µM glycine), before and 60 s after pre-treatment with 1 mM choline. Drugs were applied for 60 s, at the end of the wash out of the previous application and the arrows identify the peaks. **(B)** Fluorescence image of a field of FURA- 2-labelled synaptosomes including terminals 1–4. **(C)** Immunocytochemical co-localization of α7-nAChR, vGLUT and GluN1 in terminal 1. **(D)** Average co-localization of α7-nAChR, vGLUT and GluN1 in nerve terminals from the rat NAc. Values are mean ± S.E.M of at least four experiments.

We next carried out an immunocytochemical characterization of NAc nerve endings to gauge the extent of the co-localization between α7 nAChR and NMDAR in glutamatergic nerve terminals. As shown in Figure 3, we identified individual nerve terminals (e.g., terminal 1) that were glutamatergic (vGluT1-positive) and endowed with both GluN1 and α7 subunits (Figures 3B,C), where the pre-treatment with choline (1 mM) potentiated the NMDA (100 µM)-induced calcium transient (Figure 3A). In fact, this analysis revealed that more than 40% of glutamatergic nerve terminals (vGluT1-positive) possessed GluN1 and α7 subunits (Figure 3D), thus confirming that the co-localization of NMDAR and α7 nAChR on the same glutamatergic terminal is a generalized feature in the NAc. The analysis of individual NAc terminals further revealed non-glutamatergic (vGluT1-negative) NAc terminals (e.g., terminal 2) containing both GluN1 and α7 subunits (Figures 3B,C), where choline (1 mM) failed to modify the NMDA (100 µM)-induced calcium transient (Figure 3A). We also found terminals that responded only to the α7 nAChR agonist (e.g., terminal 3 in Figure 3A) or to NMDA (e.g., terminal 4 in Figure 3A).

**Figure 4:**
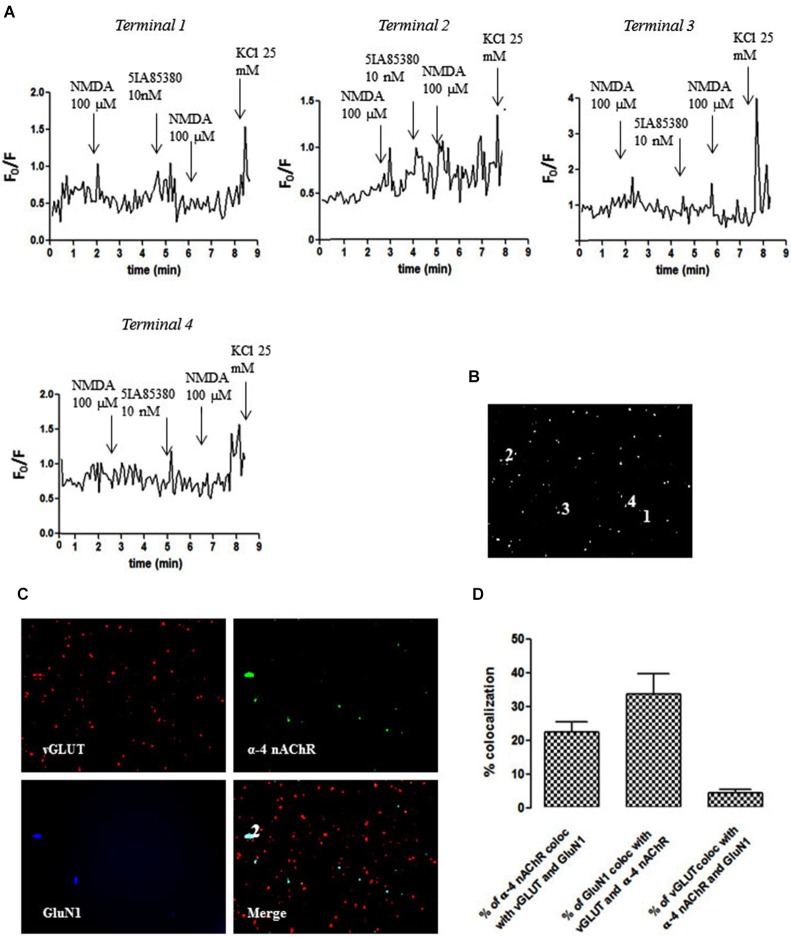
**Different impact of 5IA85380 (10 nM) pre-treatment on the ability of NMDAR agonists to trigger calcium transients in different individual terminals from the rat NAc**. **(A)** Time course of FURA-2 fluorescence emission in different individual nerve terminals (terminal 1–terminal 4), which were challenged twice with NMDAR agonists (100 µM NMDA and 10 µM glycine), before and 60 s after pre-treatment with 5IA85380 (10 nM). Drugs were applied for 60 s, at the end of the wash out of the previous application and the arrows identify the peaks. **(B)** Fluorescence image of a field of FURA-2-labelled synaptosomes including terminals 1–4. **(C)** Immunocytochemical co-localization of α4-nAChR, vGLUT and GluN1 in terminal 1. **(D)** Average co-localization of α4-nAChR, vGLUT and GluN1 in nerve terminals from the rat NAc. Values are mean ± S.E.M of at least four experiments.

We also identified individual glutamatergic nerve terminals (vGluT1-positive) containing both GluN1 and α4 subunits (terminal 2; Figures 4B,C), where the pre-treatment with 5IA85380 (10 nM) did not modify the NMDA (100 µM)-induced calcium transient (Figure 4A). Interestingly, we found other terminals (e.g., terminal 1) also containing both GluN1 and α4 subunits, where the pre-treatment with 5IA85380 (10 nM) actually reduced the NMDA (100 µM)-induced calcium transients (Figure 4A), a phenomenon previously observed in dopaminergic NAc terminals (Salamone et al., 2014). Additionally, we also observed nerve terminals responding only to NMDA (e.g., terminal 3) or to an α4 nAChR agonist (e.g., terminal 4 in Figure 4A). The average co-localization betweenGluN1 and α4 subunits (Figure 4D) showed that only 3–4% of the NAc glutamatergic nerve endings were endowed with both subunits, in contrast to the frequent co-localization of GluN1 and α7 subunits (Figure 3D).

### Pharmacological characterization of NMDAR present in NAc glutamatergic terminals

**Figure 5:**
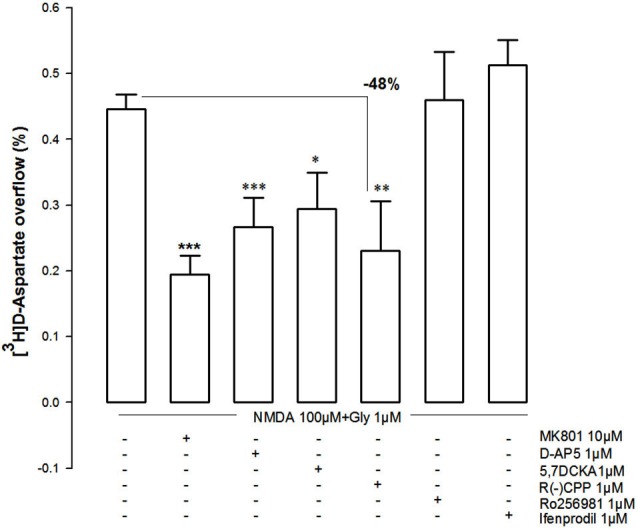
**Effect of different NMDAR antagonists on the evoked [^3^H]D-Asp release from rat NAc synaptosomes triggered by 100 µM NMDA and 1 µM glycine and lack of effect of GluN2B-NMDAR antagonists (Ro256981 and ifenprodil)**. Data are mean ± SEM of at least five experiments run in triplicate. *p < 0.05, **p < 0.01, ***p < 0.001 vs. 100 µM NMDA using a one-way ANOVA followed by Tukey-Kramer *post hoc* test.

The pharmacological characterization of the NMDAR involved in the NMDA (100 µM)-evoked [^3^H]D-Asp outflow from NAc synaptosomes is presented in Figure 5. The NMDA (100 µM)-evoked [^3^H-]D-Asp outflow was antagonized by MK801 (10 µM) and by D-AP5 (1 µM), as well as by the selective GluN1 antagonist 5,7-DCKA (1 µM). Furthermore, the GluN2A-preferring antagonist (R)-CPP (1 µM) also attenuated the NMDA (100 µM)-evoked [^3^H]D-Asp outflow (−48%), while the GluN2B-selective antagonists Ro256981 (1 µM) and ifenprodil (1 µM) were ineffective.

### nAChR activation drives GluN2A trafficking to the plasma membrane

**Figure 6:**
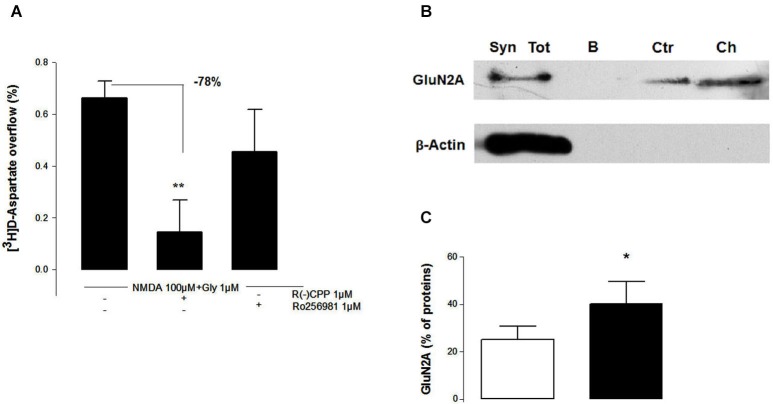
**Nicotinic acetylcholine receptors activation selectively bolsters GluN2A-dependent [^3^H]D-Asp release **(A)** and GluN2A membrane insertion **(B, C)** in NAc terminals**. **(A)** The selective GluN2A-NMDAR antagonist R(-)CPP, but not the GluN2B-NMDAR antagonist Ro256981, attenuated the potentiating effect resulting from the pre-treatment for 10 min with (1 mM Choline) of the evoked [^3^H]D-Asp release from rat NAc synaptosomes triggered by 100 µM NMDA and 10 µM glycine. Values are mean ± SEM of six experiments run in triplicate. **p < 0.01 vs. control using a one-way ANOVA followed by Tukey-Kramer *post hoc* test. **(B)** Representative Western blot of GluN2A subunit surface density in NAc terminals. The Western blots compares total synaptosomal membranes before adding biotin (Syn Tot), synaptosomal membranes that are not treated with biotin and are subject to a streptavidin pull-down **(B)**, synaptosomal membranes incubated with biotin and subject to a streptavidin pull-down (Ctr) and membranes from synaptosomes that were pre-treated for 1 mM choline before incubation with biotin and pull-down with streptavidin (Ch). The blots are representative of four different experiments carried out with synaptosomal preparations from different rats. **(C)** Comparison of the average density of biotin-labelled GluN2A proteins in NAc synaptosomal membranes without (open bars) and after (filled bars) a 10 min exposure to 100 µM nicotine. Values are mean ± SEM of four experiments. *p < 0.05 using a paired Student’s *t* test.

We next tested whether nicotine pre-treatment selectively impacts this NR2A-mediated component of the NMDA-evoked [^3^H]D-Asp outflow. As shown in Figure 6A, after (Choline 1 mM) pre-treatment, the inhibitory effect of the NR2A-preferring antagonist (R)-CPP (1 µM) was significantly increased (−78%) compared to the effects on control (non-pre-treated) synaptosomes (−48%; Figure 5). By contrast, nicotine pre-treatment did not enhance the inhibition caused by the NR2B-selective antagonist Ro256981 (1 µM), which was still non-significant (Figure 6A).

Since we have previously shown that nAChR can control the responses of presynaptic ionotropic glutamate receptors through the regulation of their trafficking in and out of the plasma membrane (Grilli et al., 2012; Salamone et al., 2014), we posited that the nicotine-induced increase of the NMDA response in NAc glutamatergic terminals would also rely on a control of the trafficking of GluN2A-containing NMDAR. Indeed, the quantification of the density of biotin-tagged GluN2A subunit proteins in NAc synaptosomes before and after choline pre-treatment (Figures 6B,C) showed that choline (1 mM) pre-treatment for 10 min increased (+15%, Figure 6C) the density of GluN2A at the plasma membrane (Figure 6B, lane Ch) respect to control (Figure 6B, lane Ctr).

### Choline potentiates the NMDA-induced D-Asp release from hippocampal nerve terminals

**Figure 7:**
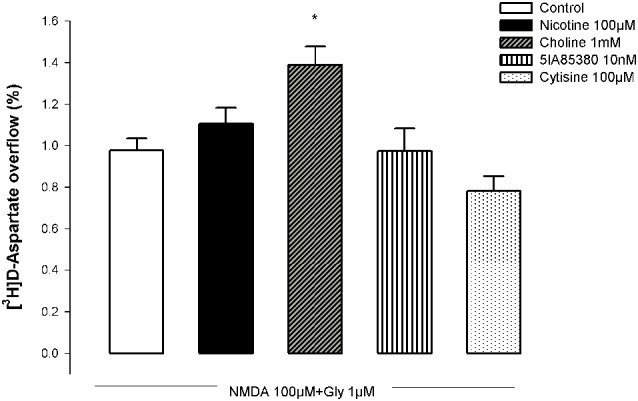
**Impact of the pre-treatment with different nAChR agonists on the ability of NMDA to trigger [^3^H]D-Asp outflow from nerve terminals of the rat hippocampus**. Each nAChR agonist was added 10 min before challenging with NMDA. Values are mean ± SEM of five experiments run in triplicate. * *p* < 0.05 vs. control.

N-methyl-D-aspartic acid (100 µM, plus 10 µM glycine) caused a marked outflow of [^3^H]D-Asp from pre-labeled hippocampal synaptosomes (Figure 7), which was quantitatively higher than that observed in NAc synaptosomes (cf. Figures 1, 7). The pre-exposure of hippocampal synaptosomes to choline (1 mM) for 10 min significantly potentiated the NMDA-induced [^3^H]D-Asp outflow while the pre-incubation with nicotine (100 µM) was ineffective. As observed in NAc synaptosomes, the pre-exposure of hippocampal synaptosomes to the α4β2 nAChR agonists 5IA85380 (10 nM) or cytisine (100 µM) for 10 min did not modify the NMDA-induced [^3^H]D-Asp outflow.

## Discussion

The present study shows that the activation of nAChR enhances the ability of NMDAR to trigger neurotransmitter release from glutamatergic terminals of the NAc. Our combined pharmacological and immunocytochemical characterization at the individual nerve terminal level revealed that this involved the ability of α 7-containing nAChR to selectively bolster GluN2A-containing NMDA receptor function. Further biochemical studies showed that nAChR activation enhanced the plasma membrane levels of GluN2A subunits in NAc terminals, allowing to argue that the nAChR-mediated control of GluN2A trafficking into the plasma membrane underlies the potentiation of presynaptic NMDAR-mediated actions by nAChR activation in NAc glutamatergic terminals.

Although ionotropic receptors are traditionally recognized as supporting fast synaptic transmission by acting as postsynaptic sensors of released neurotransmitters, evidence accumulated over the last decades also supports a parallel fine-tuning neuromodulation role for ionotropic receptors as controllers of the release of different neurotransmitters (MacDermott et al., 1999; Dorostkar and Boehm, 2008), with critical impact on adaptive changes of synaptic efficiency (Sjöström et al., 2003; Corlew et al., 2008; Bidoret et al., 2009). Accordingly, it has been shown that different nAChR and NMDAR subtypes are present in glutamatergic nerve terminals in different brain areas, where they efficiently modulate the release of glutamate (McGehee et al., 1995; Marchi et al., 2002; Bardoni et al., 2004; Dickinson et al., 2008; Musante et al., 2011; Gomez-Varela and Berg, 2013). The present study provides an additional layer of complexity in the presynaptic signaling by ionotropic receptors, dwelling on the interaction between presynaptic ionotropic receptors. In fact, building on the observation that different ionotropic receptors are co-localized in nerve terminals, we explored the nature of their interactions to grasp the fine-tuning of neurotransmitter release. Thus, our immunocytochemical findings showed that both α7 and α4 nAChR were co-localized with GluN1 subunits of NMDAR in NAc nerve terminals, namely in glutamatergic nerve endings. This led to the key observation that the two modulation systems are actually engaged in a cross-talk, since the pre-treatment of NAc synaptosomes with nicotine caused a significant increase of the NMDA-evoked intra-terminal cytosolic free calcium transient and [^3^H]D-Asp outflow.

It has been previously described that glutamate exocytosis is controlled by α7-nAChR and by α4β2-nAChR subtypes (Dickinson et al., 2008; Zappettini et al., 2010). However, our pharmacological characterization showed a primary involvement of α7-nAChR controlling presynaptic NMDA responses, based on the effects of the α7-nAChR-selective agonist choline and α7-nAChR-selective antagonist α-bungarotoxin. This is further confirmed by the lack of effect of 5IA85380, indicating the inability α4β2-nAChR to modify the functional response of presynaptic NMDAR. This contention is further strengthened by our observation that nicotine or choline triggered an increase of the NMDA-induced intra-terminal calcium transients selectively in glutamatergic nerve endings (see Figure 3), which were also endowed with α7-nAChR. Notably, the impact of nAChR activation was qualitatively similar and displayed a similar pharmacology when measuring the NMDA-induced intra-terminal calcium transients or the release of [^3^H]D-Asp. This strongly suggests that the increased NMDA-evoked outflow of glutamate probably results from the modulation of the calcium transient. Furthermore, it should be noted that α4-nAChR are also present in glutamatergic terminals (see Figure 4) and can trigger calcium entry into nerve terminals (Dickinson et al., 2007; Zappettini et al., 2010). However, α7-nAChR triggers a direct calcium entry, whereas the α4-nAChR-mediated increase of intra-terminal free calcium levels involves a depolarization of the terminal and the subsequent activation of voltage-sensitive calcium channels (Dickinson et al., 2007). This prompts the hypothesis that the different mechanisms of nAChR-induced raise of intra-terminal free calcium may be linked to their different ability to control presynaptic NMDAR function, a question that remains to be solved.

The pharmacological characterization of the nAChR-mediated control of presynaptic NMDAR responses also allowed establishing the selective involvement of GluN2A-containing NMDAR, in spite of the known presence of both GluN2A and GluN2B subunits in NMDA autoreceptors located in hippocampal glutamatergic nerve endings (Luccini et al., 2007). In fact, the NMDA-induced outflow of [^3^H]D-Asp was selectively attenuated by selective antagonists of GluN2A-containing NMDAR, whereas selective GluN2B antagonists were devoid of effects. Additionally, the pre-activation of nAChR selectively bolstered the amplitude of the inhibitory effect of GluN2A antagonists, rather than that of GluN2B antagonists, further indicating the selective nAChR modulation of presynaptic GluN2A-containing NMDAR. This was further re-enforced by the biochemical identification of an increased density of GluN2A subunits in the plasma membrane of NAc terminals after pre-activation of nAChR. This poses the control of the trafficking of NMDAR subunits as the likely mechanism operated by nAChR to bolster the effects of presynaptic NMDAR, whereas a possible impact on the exocytotic machinery is made unlikely by the lack of effect of α7-nAChR activation on the 4AP-evoked [^3^H]D-Asp outflow. Although the intracellular pathway operated by nAChR to control GluN2A trafficking remains to be defined, this might involve a nAChR-mediated control of kinase activity, since NMDAR trafficking is regulated by phosphorylation (Lan et al., 2001; Chen and Roche, 2007; Lau and Zukin, 2007).

We have previously reported that nAChR also controlled NMDAR-mediated responses in NAc dopaminergic terminals, but we found that nAChR activation depressed presynaptic NMDAR-mediated responses (Salamone et al., 2014), in contrast to the potentiation observed in NAc glutamatergic terminals and described above. Remarkable, in NAc dopaminergic nerve terminals, we observed that it was the activation of α4β2-nAChR that depressed GluN2B containing NMDAR (Salamone et al., 2014), instead of α7-nAChR potentiating GluN2A containing NMDAR in NAc glutamatergic terminals. Taken together, these findings indicate a striking difference between the interplay of nAChR and NMDAR in different nerve terminals, which seems to depend on the types of nAChR and of NMDAR playing the prime role in each different type of nerve terminal within the NAc. This prompted us to test if there were also differences between brain areas and we found that nAChR activation also triggered a potentiation of NMDAR-induced release of [^3^H]D-Asp from hippocampal nerve terminals, as occurred in the NAc glutamatergic terminals. It still remains to understand the signaling mechanisms responsible for the different setup of nAChRs and NMDARs in different types of nerve terminals in the brain.

There is increasing recognition of the importance of presynaptic NMDAR on the control of synaptic plasticity (Sjöström et al., 2003; Corlew et al., 2008; Bidoret et al., 2009), together with the role that adaptive changes in the efficiency of glutamatergic synapses may have in the addictive behavior (Ma et al., 2009; Kalivas and Volkow, 2011; Grueter et al., 2012). We characterized the ability of nAChRs to bolster presynaptic NMDAR-mediated responses in NAc glutamatergic terminals. This nAChRs-mediated control of NMDAR function in glutamatergic terminals of the NAc could help to understand the parallel effects of cholinergic and glutamatergic systems on higher brain functions involving information processing in NAc circuits such as mood, memory or addiction (Carlezon and Thomas, 2009; Reissner and Kalivas, 2010).

## Author contributions

Stefania Zappettini, performed calcium imaging analysis, immunocytochemical experiments and release experiments, revised critically the paper and approved the final version; Massimo Grilli contributed to the design of the work, coordinated and performed the release experiments, revised critically the paper and approved the final version, Guendalina Olivero, Jiayang Chen and Cristina Padolecchia performed the release experiments and revised critically the paper and approved the final version; Anna Pittaluga contributed to the design of the work, revised critically the paper and approved the final version; Angelo R. Tomé and Rodrigo A. Cunha contributed to the design of the work and coordinated the calcium imaging analysis and immunocytochemical experiments, revised critically the paper and approved the final version, Mario Marchi provided a substantial contributions to the design of the work and to the interpretation of data and wrote the paper.

## Conflict of interest statement

The authors declare that the research was conducted in the absence of any commercial or financial relationships that could be construed as a potential conflict of interest.

## Abbreviations

ECL: enhanced chemiluminescence
NAc: nucleus accumbens
nAChR: nicotinic acetylcholine receptors
NMDAR: N-methyl-D-aspartate receptor
PBS: phosphate-buffered saline
BSA: bovine serum albumin
*t*-TBS: Tris-buffered saline-Tween
5IA85380: 5-iodo-A-85380
FURA-2AM: Fura-2-acetoxymethyl ester
DHβE: dihydro-β-erythroidine
R(-) CPP: 3-((R)-2-Carboxypiperazin-4-yl)-propyl-1-phosphonic acid
MK801: (5*R*,10*S*)-(-)-5-Methyl-10,11-dihydro-5*H*-dibenzo[*a*,*d*]cylcohepten-5,10-imine maleate
5,7-DCKA: 5,7-Dichloro-4-hydroxyquinoline-2-carboxylic acid
D-AP5: D-(-)-2-Amino-5-phosphonopentanoic acid
Ro256981: (α*R*,β*S*)-α -(4-Hydroxyphenyl)-β-methyl-4-(phenylmethyl)-1-piperidinepropanol maleate
RJR2403: (*E*)-*N*-Methyl-4-(3-pyridinyl)-3-buten-1-amine oxalate.
